# Investigation of transmembrane proteins using a computational approach

**DOI:** 10.1186/1471-2164-9-S1-S7

**Published:** 2008-03-20

**Authors:** Jack Y Yang, Mary Qu Yang, A Keith Dunker, Youping Deng, Xudong Huang

**Affiliations:** 1 Department of Radiology, Brigham and Women's Hospital and Harvard Medical School, Boston, MA 02115, USA; 2 National Human Genome Research Institute, National Institutes of Health, Bethesda, MD 20892, USA; 3 Center for Computational Biology and Bioinformatics, Indiana University Schools of Medicine and Informatics, 410 W. 10th Street, Indianapolis, IN 46202, USA; 4 Department of Biological Sciences, University of Southern Mississippi, Hattiesburg, 39406, USA

## Abstract

**Background:**

An important subfamily of membrane proteins are the transmembrane α-helical proteins, in which the membrane-spanning regions are made up of α-helices. Given the obvious biological and medical significance of these proteins, it is of tremendous practical importance to identify the location of transmembrane segments. The difficulty of inferring the secondary or tertiary structure of transmembrane proteins using experimental techniques has led to a surge of interest in applying techniques from machine learning and bioinformatics to infer secondary structure from primary structure in these proteins. We are therefore interested in determining which physicochemical properties are most useful for discriminating transmembrane segments from non-transmembrane segments in transmembrane proteins, and for discriminating intrinsically unstructured segments from intrinsically structured segments in transmembrane proteins, and in using the results of these investigations to develop classifiers to identify transmembrane segments in transmembrane proteins.

**Results:**

We determined that the most useful properties for discriminating transmembrane segments from non-transmembrane segments and for discriminating intrinsically unstructured segments from intrinsically structured segments in transmembrane proteins were hydropathy, polarity, and flexibility, and used the results of this analysis to construct classifiers to discriminate transmembrane segments from non-transmembrane segments using four classification techniques: two variants of the Self-Organizing Global Ranking algorithm, a decision tree algorithm, and a support vector machine algorithm. All four techniques exhibited good performance, with out-of-sample accuracies of approximately 75%.

**Conclusions:**

Several interesting observations emerged from our study: intrinsically unstructured segments and transmembrane segments tend to have opposite properties; transmembrane proteins appear to be much richer in intrinsically unstructured segments than other proteins; and, in approximately 70% of transmembrane proteins that contain intrinsically unstructured segments, the intrinsically unstructured segments are close to transmembrane segments.

## Background

Membrane proteins account for roughly one third of all proteins and play a crucial role in processes such as cell-to-cell signaling, transport of ions across membranes, and energy metabolism [1-3], and are a prime target for therapeutic drugs [2,4-6]. One important subfamily of membrane proteins are the transmembrane proteins, of which there are two main types:

• α-helical proteins, in which the membrane-spanning regions are made up of α-helices, and

• β-barrel proteins, in which the membrane-spanning regions are made up of β-strands.

β-barrel proteins are found mainly in the outer membrane of gram-negative bacteria, and possibly in eukaryotic organelles such as mitochondria, whereas α-helical proteins are found in eukaryotes and the inner membranes of bacteria [7].

Given the obvious biological and medical significance of transmembrane proteins, it is of tremendous practical importance to identify the location of transmembrane segments. There are difficulties with obtaining the three dimensional structure of membrane proteins using experimental techniques:

• Membrane proteins have both a hydrophilic part and a hydrophobic part, and hence are not entirely soluble in either aqueous or organic solvents; this makes them difficult to crystallize, and hence difficult to analyze using X-ray crystallography, which requires crystallization of the sample.

• Membrane proteins tend to denature upon removal from the membrane, making their three-dimensional structure difficult to analyze.

The difficulty of inferring the secondary or tertiary structure of transmembrane proteins using experimental techniques has led to a surge of interest in applying techniques from machine learning and bioinformatics to infer secondary structure from primary structure in these proteins. These include discriminant analysis [8], decision trees [9], neural networks [10-13], support vector machines [14-18], and hidden Markov models [19,20].

Another interesting class of proteins are the intrinsically unstructured proteins, proteins that need not be folded into a particular configuration to carry out their function, existing instead as dynamic ensembles in their native state [21-24]. Intrinsically unstructured proteins have been associated with a wide range of functions including molecular recognition, molecular assembly/disassembly and protein modification [21,22,25].

We are interested in investigating the physicochemical properties of various classes of protein segments. In particular, we are interested in determining which properties are useful for discriminating transmembrane segments from non-transmembrane segments in transmembrane proteins, and for discriminating intrinsically unstructured segments from intrinsically structured segments in transmembrane proteins. We are further interested in any similarities or differences in physicochemical properties across these four classes of segments. We will then apply the results of this analysis to construct classifiers to discriminate transmembrane from non-transmembrane segments in transmembrane proteins.

## Results and discussion

### Physicochemical properties

We are interested in determining which physicochemical properties are most useful for discriminating transmembrane segments from non-transmembrane segments in transmembrane proteins, and for discriminating intrinsically unstructured segments from intrinsically structured segments in transmembrane proteins. We are further interested in any similarities or differences in physicochemical properties across these four classes of segments.

Certain properties, such as hydropathy and polarity, can be measured in different ways; this results in different scales. We are also interested in determining which scales are the most effective in discriminating transmembrane segments from non-transmembrane segments, and in discriminating intrinsically unstructured from intrinsically structured segments in transmembrane proteins.

Our interest is in properties that can be easily computed given only a sequence of amino acids; we therefore considered properties that depend only on the type of each amino acid in a sequence, including:

• Hydropathy, a measure of the relative hydrophobicity of an amino acid. There are four hydropathy scales in common use – the Kyte-Doolittle [26], Eisenberg-Schwarz-Komaromy-Wall [27], Engelman-Steitz-Goldman [28], and Liu-Deber [29] scales.

• Polarity, a measure of how charge is distributed over an amino acid, affects how amino acids interact, and helps to determine protein structure. There are two polarity scales in common use—the Grantham [30] and the Zimmerman-Eleizer-Simha [31] scales.

• Flexibility, a measure of the amount to which an amino acid residue contributes to the flexibility of a protein.

• Polarizability, a measure of the extent to which positive and negative charge can be separated in the presence of an applied electric field.

• van der Waals volume, a measure of the volume occupied by an amino acid.

• Bulkiness, a measure of the volume occupied by an amino acid, is correlated with hydrophobicity [32].

• Electronic effects, a measure that takes into account steric factors, inductive effects, resonance effects, and field effects [33].

• Helicity, the propensity of an amino acid to contribute to the formation of helical structures in proteins [34].

**Figure 1:**
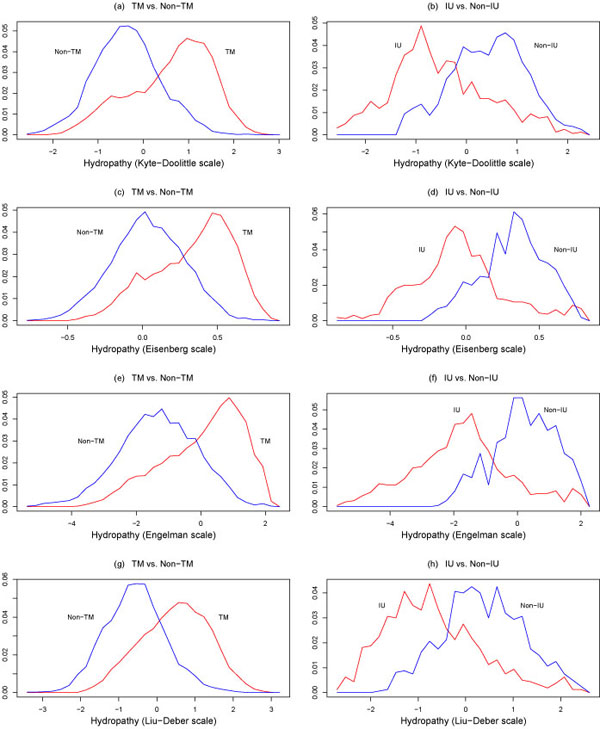
Conditional probability distributions *p*(*x*|TM), *p*(*x*|Non-TM) (on the left), and *p*(*x*|IU), *p*(*x*|Non-IU) (on the right), where *x* is hydropathy, as determined by the Kyte-Doolittle, Eisenberg-Schwarz- Komaromy-Wall, Engelman-Steitz-Goldman, and Liu-Deber scales. TM = transmembrane, IU = intrinsi-cally unstructured. The plots on the left were reproduced with permission from [38].

**Figure 2:**
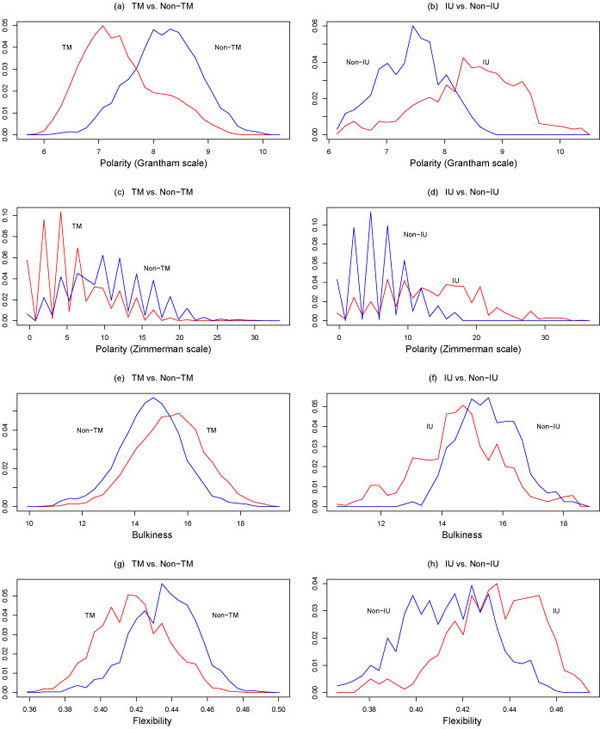
Conditional probability distributions *p*(*x*|TM), *p*(*x*|Non-TM) (on the left), and *p*(*x*|IU), *p*(*x*|Non-IU) (on the right), where *x* is, from top to bottom, polarity, as determined by the Grantham and Zimmerman-Eleizer-Simha scales, bulkiness, and flexibility. TM = transmembrane, IU = intrinsically unstructured. The plots on the left were reproduced with permission from [38].

**Figure 3:**
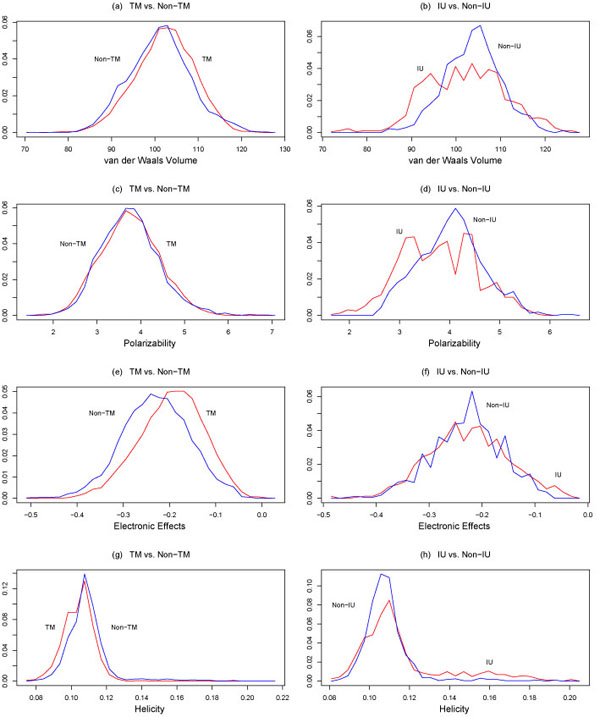
Conditional probability distributions *p*(*x*|TM), *p*(*x*|Non-TM) (on the left), and *p*(*x*|IU), *p*(*x*|Non-IU) (on the right), where *x* is, from top to bottom, van der Waals volume, polarizability, elec-tronic effects, and helicity. TM = transmembrane, IU = intrinsically unstructured. The plots on the left were reproduced with permission from [38].

Given a sequence of amino acids, the “pointwise” property value associated to a particular position in the sequence depends only on which of the 20 amino acids occurs at that position. To increase the robustness of our results, we work with average property values instead of pointwise property values. The average of a given property associated to a particular amino acid *A* in the sequence is the average of the pointwise property values associated to the amino acids contained in a window of length *L* centered at *A*. The effectiveness of each property at discriminating transmembrane from non-transmembrane segments and intrinsically unstructured from intrinsically structured segments was assessed based on two criteria:

(1) For a given property *X*, the degree to which the class-conditional distributions for the two classes overlap, that is, the degree to which *p_X_* (*x*|class 1) and *p_X_* (*x*|class 2) overlap. The less these two probability distributions overlap, the more easily the two classes can be separated. Knowledge of these probability distributions forms the basis for a Bayesian classifier, which classifies an instance having a value *x* for property *X* to “class 1” if and only if

$$\frac{px(x|class1)}{px(x|class2)}>\frac{P\{class2\}}{P\{class1\}}$$

where *P*{class 1} is the probability of observing a class 1 instance and *P*{class 2} is the probability of observing a class 2 instance. The class-conditional probability distributions for the above properties are plotted in Figures 1,2,3.

(2) The Overlap Ratio, defined in the Methods section, is a numerical measure of the overlap between the conditional probabilities P{class 1|*X* = *x*} and P{class 2|*X* = *x*}. The smaller the Overlap Ratio, the more easily the two classes can be discriminated.

The Overlap Ratios for discriminating transmembrane from non-transmembrane segments are shown in Table 1, while the Overlap Ratios for discriminating intrinsically unstructured from intrinsically structured segments are shown in Table 2. It turns out that the discriminating power of a given property depends on the length *L* of the window over which property values are averaged; Overlap Ratios are given in Tables 1 and 2 for all odd values of the window length *L* between 9 and 31.

**Table 1:** Overlap Ratios for discriminating transmembrane segments from non-transmembrane segments in membrane proteins as a function of window length (W.L.).

W.L.	*H_KD_*	*H_Ei_*	*H_En_*	*H_LD_*	***P_G_***	***P_Z_***	Bulk.	Flex.	Elec.
31	0.249	0.221	0.260	0.198	0.249	0.211	0.423	0.294	0.504
29	0.232	0.197	0.241	0.183	0.223	0.223	0.397	0.278	0.499
27	0.231	0.203	0.213	0.194	0.232	0.232	0.412	0.266	0.462
25	0.238	0.198	0.227	0.178	0.215	0.269	0.393	0.269	0.411
23	0.217	0.204	0.219	0.177	0.208	0.233	0.385	0.258	0.434
21	0.209	0.204	0.215	0.166	0.216	0.197	0.370	0.252	0.379
19	0.214	0.222	0.220	0.199	0.224	0.235	0.415	0.259	0.389
17	0.201	0.252	0.218	0.199	0.219	0.206	0.393	0.259	0.442
15	0.191	0.195	0.201	0.214	0.224	0.193	0.356	0.283	0.456
13	0.216	0.203	0.217	0.178	0.203	0.189	0.325	0.283	0.500
11	0.210	0.199	0.228	0.185	0.204	0.168	0.346	0.277	0.493
9	0.231	0.205	0.222	0.200	0.232	0.280	0.396	0.299	0.562

Here *H_KD_, H_Ei_, H_En_, H_LD_* indicate the Kyte-Doolittle, Eisenberg-Schwarz-Komaromy-Wall, Engelman-Steitz-Goldman, and Liu-Deber hydropathy scales, respectively, *P_G_, P_z_* indicate the Grantham and Zimmerman-Eliezer-Simha polarity scales, respectively, Bulk. = bulkiness, Flex. = flexibility, and Elec. = electronic effects.

Reproduced with permission from [38]

**Table 2:** Overlap Ratios for discriminating intrinsically unstructured segments from intrinsically structured segments in membrane proteins as a function of window length (W.L.).

W.L.	*H_KD_*	*H_Ei_*	*H_En_*	*H_LD_*	*P_G_*	*P_Z_*	Bulk.	Flex.
31	0.318	0.163	0.170	0.243	0.220	0.134	0.349	0.227
29	0.221	0.229	0.167	0.249	0.138	0.161	0.351	0.238
27	0.222	0.150	0.164	0.230	0.170	0.142	0.221	0.263
25	0.216	0.234	0.162	0.241	0.175	0.142	0.364	0.272
23	0.253	0.143	0.160	0.253	0.163	0.157	0.238	0.254
21	0.182	0.139	0.144	0.267	0.176	0.159	0.323	0.271
19	0.285	0.142	0.149	0.257	0.172	0.251	0.337	0.291
17	0.290	0.199	0.148	0.266	0.183	0.307	0.353	0.279
15	0.320	0.170	0.155	0.274	0.182	0.183	0.338	0.361
13	0.264	0.180	0.165	0.284	0.194	0.254	0.358	0.340
11	0.310	0.228	0.195	0.281	0.220	0.446	0.345	0.358
9	0.372	0.230	0.226	0.325	0.269	0.251	0.416	0.401

Here *H_KD_, H_Ei_, H_En_, H_LD_* indicate the Kyte-Doolittle, Eisenberg-Schwarz-Komaromy-Wall, Engelman-Steitz-Goldman, and Liu-Deber hydropathy scales, respectively, *P_G_, P_Z_* indicate the Grantham and Zimmerman-Eliezer-Simha polarity scales, respectively, Bulk. = bulkiness, and Flex. = flexibility.

Reproduced with permission from [38]

Our conclusions were as follows:

• Whereas all four hydropathy scales can be used for discriminating transmembrane segments for non-transmembrane segments in transmembrane proteins, the Liu-Deber scale is the best scale for this task.

• Whereas all four hydropathy scales can be used for discriminating intrinsically unstructured segments from intrinsically structured segments in transmembrane proteins, the Eisenberg-Schwarz-Komaromy-Wall scale is the best scale for this task.

• Whereas both polarity scales can be used for discriminating transmembrane from non-transmembrane segments and for discriminating intrinsically unstructured from intrinsically structured segments in transmembrane proteins, the Grantham scale is slightly better for these tasks.

• For both classification problems (discriminating transmembrane from non-transmembrane segments and discriminating intrinsically unstructured from intrinsically structured segments), flexibility provided some degree of discriminating power, and bulkiness provided still less; neither property was as effective as hydropathy or polarity at discriminating between the two classes.

• For both classification problems, polarizability, van der Waals volume, electronic effects, and helicity did not discriminate well between the two classes.

### Transmembrane segment classifiers

**Figure 4:**
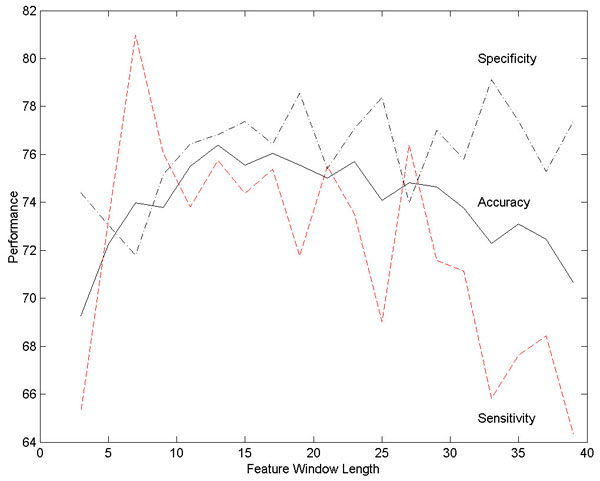
Performance of the SOGR-I classifier as a function of the length of the window used to extract features, based on threefold cross-validation (fixed learning rate η_*t*_ = .05, neighborhood size *R* = 2, number of neurons = 16). Reproduced with permission from [38].

We tested four classification techniques on the problem of discriminating transmembrane segments from non-transmembrane segments in transmembrane proteins:

• C4.5 [35], a decision tree algorithm.

• SVM^light^ version 6.01 (linear kernel function) [36], a support vector machine algorithm.

• Two variants of the Self-Organizing Global Ranking (SOGR) algorithm [37], SOGR-I [38,39] and SOGR-IB [38,39], which are described in detail in the Methods section. These algorithms depend on a number of parameters: the length *L* of the window used to extract features, the number of neurons *m*, the learning rate η_*t*_, and the neighborhood size *R*. The performance of these algorithms depends on the choice of these parameters: For example, the performance of the SOGR-I algorithm as a function of the length of the window used to extract features is shown in Figure 4. Based on a series of experiments, we settled on feature window length *L* of 10, a network size *m* of 16 neurons, a fixed learning rate η_*t*_ of .05, and a neighborhood size *R* of 2. Since the length of the window used to extract features was chosen to maximize the performance of the SOGR-I algorithm, the results will be slightly biased in favor of the SOGR-I and SOGR-IB algorithms.

Designing a classifier also involves selecting the features that are most useful for the problem of interest. Based on our investigations of physicochemical properties, we based the classification on three features:

• Hydropathy (Liu-Deber scale)

• Polarity (Grantham scale)

• Flexibility

The performance of the above four classification techniques under ten-fold cross-validation when hydropathy (Liu-Deber scale), polarity (Grantham scale), and flexibility are used as features is shown in Table 3, while the performance when only polarity (Grantham scale) and flexibility are used as features is shown in Table 4. It is interesting that performance drops only slightly when two features are used instead of three. All four classifiers exhibited good performance, with out-of-sample accuracies of approximately 75%. While this may seem low, the substantial overlap of the transmembrane and non-transmembrane classes seen in Figures 1,2,3 makes this a nontrivial classification problem. Filtering strategies can be used to improve the performance of these classifiers [38,39].

**Table 3:** Accuracy of discriminating transmembrane segments from non-transmembrane segments in trans-membrane proteins using the SOGR-I and SOGR-IB classifiers, a decision tree classifier (C4.5), and a support vector machine classifier (SVM^light^ version 6.01), based on ten-fold cross-validation. Three features were used, namely hydropathy (Liu-Deber scale), polarity (Grantham scale), and flexibility.

			C4.5		
Fold	SOGR-I	SOGR-IB	Before Pruning	After Pruning	SVM
1	72.2311	72.2311	72.4960	72.5490	72.9730
2	69.0476	67.1733	67.8318	67.6798	67.3252
3	77.1277	76.9149	77.5532	77.6596	77.7660
4	81.8913	84.5875	83.7827	83.7827	83.4608
5	79.3146	78.4889	78.3237	78.4476	78.1586
6	81.4600	83.6230	82.8119	83.1595	82.0780
7	75.9410	76.8266	75.6458	75.9410	76.3100
8	78.3488	79.2783	79.8797	79.9891	79.2783
9	64.1365	65.0418	64.5543	64.5543	64.7632
10	67.2325	65.6089	66.8635	67.0111	67.6753
Mean	74.7	75.0	75.0	75.1	75.0
Std. dev.	6.2	7.2	6.8	6.8	6.5

Reproduced with permission from [38]

**Table 4:** Accuracy of discriminating transmembrane segments from non-transmembrane segments in trans-membrane proteins using the SOGR-I and SOGR-IB classifiers, a decision tree classifier (C4.5), and a support vector machine classifier (SVM^light^ version 6.01), based on ten-fold cross-validation. Two features were used, namely polarity (Grantham scale) and flexibility.

			C4.5		
Fold	SOGR-I	SOGR-IB	Before Pruning	After Pruning	SVM
1	71.7541	72.0721	72.3900	72.6020	72.6550
2	65.1469	65.8561	66.1601	66.1601	67.0719
3	77.1277	78.4043	76.3830	77.5532	77.4468
4	83.0986	85.0302	83.7827	83.7827	83.0181
5	77.2502	77.6631	76.4244	76.4244	79.1082
6	81.9235	83.2368	82.8505	82.8119	82.1166
7	75.5720	76.6052	75.7934	75.8672	75.9410
8	79.4423	79.4423	79.7704	79.4970	79.4423
9	64.1365	64.3454	64.2061	64.2061	64.4150
10	67.4539	67.5277	67.0849	67.0849	67.0849
Mean	74.3	75.0	74.5	74.6	74.8
Std. dev.	6.8	7.2	6.9	6.9	6.7

Reproduced with permission from [38]

## Conclusions

We determined that the most useful properties for discriminating transmembrane segments from non-transmembrane segments and for discriminating intrinsically unstructured segments from intrinsically structured segments in transmembrane proteins were hydropathy, polarity, and flexibility, and based on these properties, constructed a number of classifiers to identify transmembrane segments with an out-of-sample accuracy of approximately 75%. Several interesting observations emerged from our study:

• Intrinsically unstructured segments and transmembrane segments tend to have opposite properties, as summarized in Table 5. For example, unstructured segments tended to have a low hydropathy value, whereas transmembrane segments tended to have a high hydropathy value. These results are in agreement with previous work that found that transmembrane segments tend to be more hydrophobic than non-transmembrane segments, due to the fact that transmembrane α-helices require a stretch of 12-35 hydrophobic amino acids to span the hydrophobic region inside the membrane [26].

**Table 5:** Tendencies of various properties for tranmembrane (TM) and intrinsically unstructured (IU) segments.

	Segment	Type
Property	TM	IU
Hydropathy	High	Low
Polarity	Low	High
Bulkiness	High	Low
Flexibility	Low	High
Electronic effects	High	Low

Reproduced with permission from [38]

• Transmembrane proteins appear to be much richer in intrinsically unstructured segments than other proteins; about 70% of transmembrane proteins contain intrinsically unstructured regions, as compared to about 35% of other proteins.

• In approximately 70% of transmembrane proteins that contain intrinsically unstructured segments, the intrinsically unstructured segments are close to transmembrane segments.

These observations may provide insight into the structural and functional roles that intrinsically unstructured segments play in membrane proteins, and may also aid in the identification of intrinsically unstructured and transmembrane segments from primary protein structure.

## Methods

### Physicochemical properties

The Overlap Ratio, a quantitative measure of how well two classes (referred to generically as “class 1” and “class 2”) can be discriminated based on a property *X*, was calculated as follows.

1. We construct a graph such that:

(a) The horizontal axis corresponds to the property X. We divide this axis into bins.

(b) The y-value associated with the bin corresponding to X values between *x* and *x* + ∈ is the fraction of all instances in the training set that belong to class 1 and have a value for the feature X in the range [*x*, *x* + ∈), where ∈ > 0 is small.

The graph represents an approximation to the function P{class 1|*X* = *x*}. We define the complementary function P{class 2|*X* = *x*}using

P{class 2|X=x}=1−P{class 1|X=x}

2. Let

$$\begin{array}{l} f_{1}(x)\equiv \text{P}\{\text{class }1|X=x\}\\ f_{2}(x)\equiv \text{P}\{\text{class 2}|X=x\} \end{array}$$

Then the Overlap Ratio is then defined as:

$$\text{overlap Ratio}=\frac{\text{Area under both }f_{1}(x)\text{ and }f_{2}(x)}{\text{Area under }f_{1}(x)+\text{ Area under }f_{2}(x)}$$

The smaller the Overlap Ratio, the more easily the two classes can be discriminated.

### The SOGR-I and SOGR-IB classification algorithms

#### Overview

The Self-Organizing Global Ranking (SOGR) algorithm [37] was inspired by Kohonen's Self-Organizing Map (SOM) algorithm [40]. In the SOM algorithm, each neuron has associated with it a topological neighborhood, and the algorithm is such that neighboring neurons in the topological space tend to arrange themselves over time into a grid in feature space that mimics the neighborhood structure in the topological space. The SOGR algorithm differs from the SOM algorithm by dropping the topological neighborhood and replacing it with the concept of a global neighborhood generated by ranking. We consider two variants of the SOGR algorithm:

• The first variant, SOGR-I [38,39], modifies the initialization scheme of SOGR.

• The second variant, SOGR-IB [38,39] (“B” stands for “Batch update”), removes the dependence on the order in which instances are presented by only updating the weights after each cycle, where a cycle involves presenting the entire training set to the network, one instance at a time. This variant also uses the modified initialization procedure described above.

Before we describe the above modifications in detail, we describe the SOGR algorithm itself.

#### The SOGR classification algorithm

We assume that *m* neurons are used; each neuron *j* has a weight vector ${\vec{W}}_{j}$ (*t*), where *t* represents time. Let the initial position of neuron *j* at time *t* = 0 be ${\vec{W}}_{j}$ (0), and assume that the training set consists of instances (${\vec{x}}_{i}$, *y_i_*), *i* = 1, … , *n*, where the ${\vec{x}}_{i}$ are feature vectors, and *y_i_* denotes the class of an instance.

1. **Initialization:** Choose initial positions ${\vec{W}}_{j}$ (0) in feature space for the *m* neurons by assigning the neurons random positions in feature space.

2. Present the instances in the training set to the network, one at a time. As each instance is presented to the network, the time index *t* is increased by 1. For each instance (${\vec{x}}_{i}$, *y_i_*) in the training set, the positions of one or more neurons are adjusted as follows:

• **Identifying Winning Neurons:** Find the *R* closest neurons to the feature vector ${\vec{x}}_{i}$, that is, find the *R* neurons with the smallest value of $∥{\vec{x}}_{i}\;-\;{\vec{W}}_{j}(t)∥$. These *R* neurons constitute the “neighborhood” of the input vector. Let Γ be the set of indices of the *R* winning neurons.

• **Updating Weights:** Adjust the positions of each of the *R* winning neurons using the update rule

$${\vec{W}}_{j}(t+1)={\vec{W}}_{j}(t)+\eta_{t}({\vec{x}}_{i}-{\vec{W}}_{j}(t))$$

where *j* ∈ Γ and η_*t*_ is the learning rate. The learning rate is chosen to decrease with time in order to force convergence of the algorithm. In [37] it is suggested that the learning rate be decreased at an exponential rate, and that it should be smaller for larger neighborhood sizes *R*.

3. **Assigning Classes to Neurons:** Associated with each neuron *j* is a count of the number of instances belonging to each class that are closer to neuron *j* than any other neuron. This count is calculated as follows:

• For each neuron, initialize the counts to zero.

• For each instance (${\vec{x}}_{i}$, *y_i_*) in the training set, find the closest neuron to the feature vector ${\vec{x}}_{i}$, that is, find the neuron with the index j^*^, where

$$j*=\text{arg }\underset{j}{\min}||{\vec{x}}_{i}-{\vec{W}}_{j}(t)||$$

and increment the count in neuron j^*^ corresponding to class *y_i_* by 1.

• After all instances in the training set have been considered, each neuron is assigned to the class corresponding to the largest count for that neuron.

After the training process has been completed, a test instance can be classified by assigning it the class label of the nearest neuron.

#### The SOGR-I classification algorithm

The first variant, SOGR-I [38,39], modifies the initialization scheme of SOGR. Specifically, assume that the feature space is *d* dimensional, so that the feature vectors ${\vec{x}}_{i}$ belong to ${\mathbb{R}}^{d}$. For each feature *k*, we find the largest and smallest value of that feature over the entire training set, which are respectively *L_k_* and *U_k_*:

$$\begin{array}{l} L_{k}=\underset{i}{\min}x_{ik}\\ U_{k}=\underset{i}{\min}x_{ik} \end{array}$$

where *x_ik_* is the *k^th^* element of the feature vector ${\vec{x}}_{i}$. Then the initial positions of the *m* neurons are chosen as:

$$W_{jk}(0)=L_{k}+\frac{j-1}{m-1}(U_{k}-L_{k})\;\begin{array}{cc} j= & 1,\ldots ,m\\ k= & 1,\ldots ,d \end{array}$$

Thus the *m* neurons are evenly distributed along the line connecting (*L*_1_, *L*_2_, … *L_d_*) to (*U*_1_, *U*_2_, … *U_d_*). This approach has several advantages over other initialization methods:

• It guarantees that the neurons will be in some sense evenly distributed throughout the feature space. Random initialization, on the other hand, does not guarantee this. If one has a large feature space, say of 60 dimensions, and comparatively few neurons, say 50, then with random initialization those neurons will with high probability not be evenly distributed throughout the feature space.

• Even a small number of neurons can be used to populate the feature space. If we consider an alternate initialization procedure in which one populates the feature space with a d-dimensional grid of neurons, and there are *q* grid points along each feature space axis, then the total number of neurons required to populate this grid is *q^d^*. For example, if *q* = 3 and the feature space has 60 dimensions, then the number of neurons required is

$$q^{d}=3^{60}\approx 4.239\times 10^{28}$$

which is clearly infeasible.

#### The SOGR-IB classification algorithm

The second variant, SOGR-IB [38,39], addresses two problems with the original SOGR algorithm:

• The SOGR algorithm updates the weights after each new instance is presented to the network; as a result, the neuron trajectories can oscillate wildly.

• The SOGR algorithm specifies that the learning rate should be decreased during the course of training, for example at an exponential rate. The problem is that if the learning rate is decreased too rapidly, then the neurons may get stuck before they have reached their optimal positions.

SOGR-IB (“B” stands for “Batch update”) addresses these problems in two ways:

• It uses a “batch update” strategy for updating the positions of the neurons in feature space. This eliminates the dependence of the results on the order in which instances are presented to the network, and also stabilizes the trajectories of the neurons.

• The batch update strategy allows the use of a fixed, but small, learning rate η_*t*_, which eliminates the problem of the weights getting stuck because the learning rate η_*t*_ was decreased too quickly.

The SOGR-IB algorithm is described below:

1. **Initialization:** Choose initial positions ${\vec{W}}_{j}$(0) in feature space for the *m* neurons using the SOGR-I initialization strategy. Set *t* = 0.

2. Repeat the following until the “energy” defined by

$$Q(t)\;=\;\frac{1}{2nR}\;\sum_{\text{instances }i}^{}\;\sum_{\text{neurons }j}^{}\;m_{ij}∥{\vec{x}}_{i}\;-\;{\vec{W}}_{j}(t){∥}^{2}$$

does not reach a new minimum over a number of iterations through the training set, where *n* is the number of training instances, *R* is the number of neurons neighboring a given training instance that will be updated, and for each instance (${\vec{x}}_{i}$, *y_i_*) in the training set, *m_ij_* = 1 for neurons *j* that are one of the R closest neurons to the feature vector ${\vec{x}}_{i}$, and *m_ij_* = 0 for all other neurons *j*. After each pass through the training set, the time index *t* is incremented by 1.

(a) Let ${\vec{Z}}_{j}$ be the “accumulator” corresponding to neuron *j*. Initialize ${\vec{Z}}_{j}$ to 0 for all neurons *j*.

(b) Present the instances (${\vec{x}}_{i}$, *y_i_*) in the training set to the network, one at a time. After each instance is presented, the “accumulators” are updated as follows:

• **Identifying Winning Neurons:** Find the *R* closest neurons to the feature vector ${\vec{x}}_{i}$, that is, find the *R* neurons with the smallest value of $∥{\vec{x}}_{i}\;-\;{\vec{W}}_{j}(t)∥$. These *R* neurons constitute the “neighborhood” of the input vector. Let Γ be the set of indices of the *R* winning neurons.

• **Updating Accumulators:** Adjust the accumulators corresponding to each of the *R* closest neurons using the update rule

$${\vec{Z}}_{j}\;=\;{\vec{Z}}_{j}\;+\;\frac{1}{nR}\eta_{t}({\vec{x}}_{i}-\;\vec{W}(t))$$

where *j* ∈ Γ and η_*t*_ is the learning rate.

(c). **Updating Neurons:** After all instances in the training set have been presented to the network, update the weights for each neuron *j* using the rule:

$${\vec{W}}_{j}\left(t+1\right)={\vec{W}}_{j}\left(t\right)+{\vec{Z}}_{j}$$

where *n* is the number of instances in the training set.

3. **Assigning Classes to Neurons:** Same as Step 3 in the SOGR algorithm above.

## Competing interests

The authors declare that they have no competing interests.

## Authors' contributions

JYY conceived of the project; MQY and JYY contributed ideas to the project; MQY designed the project; MQY performed the experiments and analyses, and wrote the manuscript; AKD, YPD and XH contributed suggestions.
